# Assessing the readiness of precision medicine interoperability: an exploratory study of the National Institutes of Health Genetic Testing Registry

**DOI:** 10.14236/jhi.v24i4.918

**Published:** 2017-11-17

**Authors:** Jay G. Ronquillo, Chunhua Weng, William T. Lester

**Affiliations:** Western Michigan University Homer Stryker M.D. School of Medicine, Kalamazoo, MI, USA; Columbia University, New York, NY, USA; Massachusetts General Hospital, Boston, MA, USA and Harvard Medical School, Boston, MA, USA

**Keywords:** informatics, electronic health records, genomics, interoperability, precision medicine

## Abstract

**Background:**

Precision medicine involves three major innovations currently taking place in healthcare: electronic health records, genomics and big data. A major challenge for healthcare providers is, however, understanding the readiness for the practical application of initiatives like precision medicine.

**Objective:**

To better understand the current state and challenges of precision medicine interoperability using a national genetic testing registry (GTR) as a starting point, placed in the context of established interoperability formats.

**Methods:**

We performed an exploratory analysis of the National Institutes of Health GTR. Relevant standards included Health Level Seven International Version 3 Implementation Guide for Family History, the Human Genome Organization Gene Nomenclature Committee (HGNC) database and Systematised Nomenclature of Medicine – Clinical Terms (SNOMED CT). We analysed the distribution of genetic testing laboratories, genetic test characteristics and standardised genome/clinical code mappings, stratified by laboratory setting.

**Results:**

There were a total of 25,472 genetic tests from 240 laboratories testing for approximately 3,632 distinct genes. Most tests focused on diagnosis, mutation confirmation and/or the risk assessment of germline mutations that could be passed to offspring. Genes were successfully mapped to all HGNC identifiers, but less than half of tests were mapped to SNOMED CT codes, highlighting significant gaps when linking genetic tests to standardised clinical codes that explain the medical motivations behind test ordering.

**Conclusion:**

While precision medicine could potentially transform healthcare, successful practical and clinical applications will first require the comprehensive and responsible adoption of interoperable standards, terminologies and formats across all aspects of the precision medicine pipeline.

## INTRODUCTION

Over the last few years, healthcare has been undergoing dramatic transformation due to the rapid growth of three important innovations: electronic health records (EHRs), genomics and big data. The Affordable Care Act has substantially increased the volume of patients demanding care and is transforming the way healthcare organisations must provide that care. In particular, healthcare providers will be held more accountable for their ability to meet important quality measures such as adequate control of chronic disease, broad preventive health-care and significant reductions in hospitalisations.^1^ Similarly, the Health Information Technology for Economic and Clinical Health Act has led to a dramatic increase in the adoption of EHR technology throughout the country.^2^ The development of mandated meaningful use requirements and interoperability standards has propelled the growth and availability of EHRs and other forms of health information technology (IT).^1^ This influx of new regulations and digital records will encourage large amounts of clinical and quality data to be managed, shared and applied across diverse organisations and institutions.^3^

At the same time that EHRs are providing increased access to clinical information, the sequencing of the human genome over a decade ago has catalysed new discoveries explaining the genetic contributions of a patient’s susceptibility to disease.^4^ Precision medicine is broadly defined as the application of patient-specific health and genomic information for highly targeted and effective methods of clinical diagnosis, management and treatment.^5,6^ This genomic data can come from many sources, from single and multigene tests to the sequencing of exomes and entire genomes, all of which provide valuable insight into the human clinical condition.^4^ Precision medicine has the potential to leverage health IT in ways that could dramatically improve public and population health, bringing practical genomic information exchange into sharp focus.^7^

A major challenge for healthcare providers is, however, the rapid and dramatic increase in the volume and complexity of data that must now be collected, organised and evaluated.^2^ For EHRs, the large amount of clinical information includes medical history, laboratory results, imaging files and other data collected during the patient appointment.^2^ For genomics, this involves descriptive metadata along with the sequenced genome of a patient, which (at roughly six billion base pairs in size) will require significant investments in storage, analysis and dynamic interpretation.^4^ These challenges will be compounded exponentially when scaled to the patient population affected by EHR adoption and genomics. It is widely predicted that current EHRs alone will not be capable of handling the volume and complexity of genomic information central to the practice of precision medicine.^8–10^ Indeed, no universally accepted approach exists for describing clinically relevant genomic findings, and a very few EHRs today even attempt to report both clinical and genomic data at the point of care.^11–13^ As a result, understanding the current state of precision medicine interoperability will be a key first step towards effectively annotating results and communicating genetic test information across different health IT systems.^14,15^ The goal of this study is to analyse the genetic testing registry (GTR) in the context of relevant health IT nomenclatures and standards in order to understand the practical challenges in addressing interoperability for precision medicine.

## METHODS

### Data collection, definition and classification

The National Institutes of Health (NIH) GTR provides a comprehensive description of registered genetic tests being offered by various laboratories and organisations for clinical applications.^16^ Laboratories add their genetic tests to the registry by submitting an online template with detailed information about their test and its applications. Minimum required fields include the genetic test name, purpose, laboratory information, methodology and related conditions/phenotypes.^16,17^ The test purpose required field, described in detail online, currently includes twelve indications for a genetic test (for which multiple selections are allowed): diagnosis, drug response, monitoring, mutation confirmation, pre-implantation genetic diagnosis, pre-symptomatic assessment of high-penetrance genetic disorders, risk assessment, screening, prognostic, predictive, recurrence and therapeutic management.^16,17^ A full version of the GTR data set was downloaded from the NIH GTR website.^17^ In particular, this included the complete public data set and additional files describing genetic tests, disease names, and gene-disease relationships.

Using available organisations, institutions and/or department names, laboratories that offered or performed genetic testing were classified into four categories according to the specific organisational setting in which genetic testing was performed: 1) Academic/Hospital (laboratories affiliated with a university or medical center), 2) Company (laboratories part of for-profit organisations or companies), 3) Institute/Center (laboratories affiliated with a non-academic/non-medical institute or center), and 4) Other (laboratories not falling into any prior category).

### Interoperability standards and databases

As a starting point for assessing health IT conformance to standards for exchanging genomic data, we chose the Health Level Seven International (HL7) Version 3 Implementation Guide for Family History/Pedigree Interoperability, Release 1.^18^ In particular, the minimal core data set in this standard requires mapping data to the Human Genome Organisation Gene Nomenclature Committee (HGNC) database that includes National Center for Biotechnology Information (NCBI) RefSeq identifiers. Clinical conditions targeted by various genetic tests were further described in the registry as Systematised Nomenclature of Medicine – Clinical Terms (SNOMED CT) codes.

### Analytics pipeline and statistical analysis

Using the above definitions, categories and interoperability standards, an analytical pipeline was created in the Python programming language to extract all clinical genetic test information from the NIH GTR, to map available genetic test data to standard identifiers in the HGNC database, and to create an integrated data set for analysis.^19^ Summary statistics were collected for categorical data as frequencies and percentages, with differences (laboratory setting according to mutation type, the number of genes tested, test purpose and SNOMED CT mapping status, respectively) evaluated using chi-square or Fisher’s exact test, as appropriate. A *P* value <0.05 was considered significant. Analyses were performed using R version 3.1.1 (R Foundation for Statistical Computing) and open source statistical software PSPP version 0.8.4.

## RESULTS

### Genetic testing registry characteristics

There were a total of 25,472 genetic tests from 240 different laboratories in the NIH GTR, testing for approximately 3,632 distinct genes. Of these tests, 23,999 (94.2%) were submitted directly to the NIH GTR, while 1,473 (6.1%) carried over from the prior GeneTests Laboratory Directory. The distribution of laboratory categories included 125 (52.1%) laboratories in the Academic/Hospital setting, 65 (27.1%) affiliated with a Company, 28 (11.7%) from an Institute/ Center and 22 (9.2%) in Other. Multiple genes were evaluated in 1,933 (8.1%) tests, often as part of panels that may use next-generation sequencing methods, while most of the remaining 22,066 (92.0%) genetic tests submitted directly to the registry focused on evaluating or assessing single genes (Table 1). Further, 23,829 (99.3%) tests focused on germline mutations, 113 (0.5%) on somatic mutations and 57 (0.2%) did not provide this information (Table 1). On average, there were 50.5 tests per Academic/Hospital site, 206.7 tests per Company site, 94.1 tests per Institute/Center site and 73.5 tests per Other site.

### Purpose of genetic testing

The reported purposes for each genetic test were as follows: 23,274 (97.0%) diagnosis, 147 (0.6%) drug response, 384 (1.6%) monitoring, 11,119 (46.3%) mutation confirmation, 151 (0.6%) pre-implantation diagnosis, 5,354 (22.3%) pre-symptomatic, 609 (2.5%) predictive, 146 (0.6%) prognostic, 29 (0.1%) recurrence, 5,668 (23.6%) risk assessment, 4,741 (19.8%) screening and 154 (0.6%) therapeutic management. By laboratory setting (Table 2), the top three reported purposes by test volume were diagnosis (6,122), mutation confirmation (4,418) and risk assessment (3,343) for Academic/ Hospital; diagnosis (13,055), mutation confirmation (5,260) and pre-symptomatic (2,083) for Company; diagnosis (2,631), mutation confirmation (855) and risk assessment (528) for Institute/Center; and diagnosis (1,466), mutation confirmation (586) and pre-symptomatic (345) for Other.

### Interoperability mappings related to precision medicine

All 3,632 genes could be matched to an approved gene symbol from HGNC, and 3,594 (99.0%) of these genes were successfully mapped to NCBI RefSeq identifiers. There were a total of 5,318 conditions associated with these tests, with 1,163 (21.9%) conditions in the registry currently assigned a SNOMED CT code. Out of 1,160 SNOMED CT codes, 146 (12.6%) mapped to a single genetic test, while 1,014 (87.4%) mapped to multiple genetic tests. Alternatively, the extent that genetic tests in the NIH GTR could be mapped to one or more SNOMED CT codes, as shown in Table 3. Furthermore, tests in both the Institute/Center and Other laboratory settings mapped to as many as ten different SNOMED CT codes, while some genetic tests in the Academic/Hospital (e.g. Baylor MitoMet Plus aCGH analysis) and Company (e.g. Illumina TruGenome Predisposition Screen) settings mapped to over 250 and 500 SNOMED CT codes, respectively.

## DISCUSSION

Precision medicine is expected to play a key role in transforming healthcare, and interoperable health IT provides the critical infrastructure around which precision medicine can be applied. To the author’s knowledge, this is the first study to assess the current state of precision medicine interoperability by analyzing GTR data with existing interoperability standards. This study is timely given the announced U.S. Precision Medicine Initiative (PMI), also known as the NIH *All of Us* Research Program, and the rapid convergence of health IT, genomics and big data analytics.^7,20,21^

There were a large number of registered genetic tests for a diverse set of genes focused primarily on diagnosis, mutation confirmation and/or risk assessment. When broken down by laboratory setting, academic institutions focused primarily on the diagnosis or confirmation of mutations, while companies reported a much more diverse set of registered purposes. This likely reflects the differing priorities and varied stakeholders involved for these settings. Companies, for example, develop tests for a broad set of stakeholders (including directly to consumers) consistent with the diverse reported test purposes, while tests at academic hospitals focus heavily on helping physicians, addressing the clinical diagnostic needs of their patient populations.^22^

While tests for germline mutations that could be passed to offspring predominated, the expansion of registry submission criteria will likely lead to a growing volume of genetic tests for somatic mutations as well.^16^ The relatively small volume of tested genes in the registry likely reflects the current lack of evidence supporting the clinical validity and utility of most genes in the human genome; furthermore, unlike analytical validity, clinical validity and utility remain optional entries in the NIH GTR.^2,16^ Even at this early stage of precision medicine, however, several laboratories have begun offering genomic sequencing services and evaluating large panels of genes.^17^ Oncology is an example of one important area for precision medicine, and where an understanding of the human genome has guided not only disease risk assessment and diagnosis, but also selection of the most effective treatments for patients as well.^7,23,24^ As new guidelines and standards for identifying, classifying and assessing evidence for genomic data are developed, the breadth of clinically relevant genes will likely expand considerably over time.^25,26^

The successful application of precision medicine in prac-tice will require health IT capable of processing large volumes of genomic data and presenting relevant results to physicians at the point of care.^2,4,27^ While the largest number of laboratories came from the Academic/Hospital setting, the largest volume of actual tests originated from companies; in particular, there were twice as many academic/hospital labs than company laboratories, yet those companies registered twice as many genetic tests. Prior studies have shown different types of physicians order different genetic tests, and this study similarly showed that different organisations focus on different types of assays.^22^ Effective practical adoption of precision medicine will require a strong understanding of the diverse backgrounds and behaviours of stakeholders, ranging from patients being tested to providers ordering tests to the labs building new technologies.^22,28^

One major purpose of the NIH GTR is to help healthcare providers to make informed decisions about the need to order genetic tests for patients.^16^ Genomic clinical decision support largely depends on the ability to connect genetic information with relevant clinical conditions at the point of care.^27,28^ While the majority of genes were successfully mapped to both HGNC-approved gene symbols and NCBI RefSeq identifiers, a majority of genetic tests did not have any SNOMED CT code assigned to them, reflecting a critical gap in core information needed for the practice of precision medicine. The voluntary nature of the GTR is likely a major contributor to the poor degree of clinical mapping. In particular, not only is submission to the NIH GTR optional for organisations, but critical data fields (e.g. clinical codes, clinical validity and clinical utility) are currently the optional components of each submission as well. A required mapping of medical and clinical terms through a mandatory registry submission process would make the NIH GTR a more valuable resource to help physicians make sense of the overwhelming volume of genomic information that may soon be integrated into clinical care.^2,4,27,29^

Currently, multiple genetic tests map to a single SNOMED CT code, obligating physicians to spend time deciding among multiple options for the same clinical indication. The presence of incomplete or confusing clinical mappings for genetic tests is likely due to current uncertainty around which standards should be used to map genetic data with other types of medical information.^29–31^ The U.S. Federal Government’s Precision Medicine Task Force, for example, is responsible for recommending the set of standards to be used for exchanging data for the million (or more) patients expected to participate in the National PMI. Yet even with hundreds of relevant standards available, from Fast Healthcare Interoperability Resources to HL7 Clinical Genomics standards to the Global Alliance for Genomic Health, surprisingly only one standard (HL7’s Family Health History/Pedigree) has been recognised by the task force as mature enough for practical use in the PMI.^32,33^ Strong multidisciplinary leadership capable of addressing the critical technical, regulatory and interoperability gaps will be needed so that the vision of precision medicine can become a practical reality.

Our study had several limitations. First, the results may not be generalisable since the GTR is a voluntary registry that may not capture every laboratory offering genetic testing services, and selection bias is thus possible. However, this NIH-based registry currently represents the most comprehensive attempt at creating a centralised resource of genetic tests and laboratories for the healthcare community, and will likely become more complete over time with the growing focus on precision medicine.^16^ Second, the dynamic nature of genomic medicine means that any categories used in our study to describe genetic tests will likely change as the field evolves. However, our study provides a solid starting point for gaining useful insight into the current state of precision medicine, and language describing the field will begin to stabilise as standards are adopted, guidelines are developed and policies and regulations are put in place.^7,31,34–36^ Finally, there is a wealth of available standards and formats that could be applied to precision medicine, but the analysis of any single standard would not be able to adequately address every major issue. The primary purpose of our study was to take a data-driven approach to assessing the challenges and opportunities of precision medicine through the lens of health IT interoperability. As precision medicine evolves from assessing genetic tests to applying sequenced genomes, informatics approaches can be used to provide valuable insight into the wealth of diverse data describing all aspects of health-care IT.^27^

In conclusion, the practice of precision medicine enabled by interoperable health IT has the potential to dramatically improve healthcare. However, this will first require the comprehensive but responsible adoption and implementation of appropriate standards, terminologies and formats across all aspects of the precision medicine pipeline.

## Figures and Tables

**Table 1:** Genetic test characteristics by laboratory setting

Test characteristic	Academic/Hospital (N = 6312)	Company (N = 13437)	Institute/Center (N = 2634)	Other (N = 1616)
Mutation type, No (%)^a^				
Germline	6282 (99.5)	13315 (99.1)	2625 (99.7)	1607 (99.4)
Somatic	28 (0.4)	68 (0.5)	8 (0.3)	9 (0.6)
Unknown	2 (0.0)	54 (0.4)	1 (0.0)	0 (0.0)
Genes tested, No (%)^b^				
Single gene	5772 (91.4)	12431 (92.5)	2503 (95.0)	1360 (84.2)
Multiple genes	540 (8.6)	1006 (7.5)	131 (5.0)	256 (15.8)

a p < 0.001 comparing distribution of mutation types by Laboratory setting

b p < 0.001 comparing distribution of genes number by Laboratory setting

**Table 2:** Registry-reported test purposes by laboratory setting

Purpose(s) of test, No (%)^a^^,^^b^	Academic/Hospital (N = 6312)	Company (N = 13437)	Institute/Center (N = 2634)	Other (N = 1616)
Diagnosis	6122 (97.0)	13055 (97.2)	2631 (99.9)	1466 (90.7)
Drug Response	63 (1.0)	61 (0.5)	11 (0.4)	12 (0.7)
Monitoring	360 (5.7)	18 (0.1)	5 (0.2)	1 (0.1)
Mutation Confirmation	4418 (70.0)	5260 (39.1)	855 (32.5)	586 (36.3)
Pre-implantation diagnosis	103 (1.6)	7 (0.1)	23 (0.9)	18 (1.1)
Pre-symptomatic	2828 (44.8)	2083 (15.5)	98 (3.7)	345 (21.3)
Predictive	29 (0.5)	530 (3.9)	10 (0.4)	40 (2.5)
Prognostic	93 (1.5)	37 (0.3)	12 (0.5)	4 (0.2)
Recurrence	5 (0.1)	5 (0.0)	1 (0.0)	18 (1.1)
Risk Assessment	3343 (53.0)	1637 (12.2)	528 (20.0)	160 (9.9)
Screening	2138 (33.9)	2010 (15.0)	415 (15.8)	178 (11.0)
Therapeutic Management	18 (0.3)	126 (0.9)	8 (0.3)	2 (0.1)

a Percentages do not sum to 100 since multiple purposes can be reported for tests

b p < 0.001 comparing distribution of test purpose by laboratory setting

**Table 3:** Mapping of tests to clinical codes by laboratory setting

Tests mapped to SNOMED codes, No (%)^a^	Academic/Hospital (N = 6312)	Company (N = 13437)	Institute/Center (N = 2634)	Other (N = 1616)	Total (N = 23999)
Mapped					
Single code	2419 (38.3)	4550 (33.9)	1082 (41.1)	603 (37.3)	8654 (36.1)
Multiple codes	558 (8.8)	766 (5.7)	102 (3.9)	132 (8.2)	1558 (6.5)
Single/multiple codes	2977 (47.2)	5316 (39.6)	1184 (45.0)	735 (45.5)	10212 (42.6)
Unmapped	3335 (52.8)	8121 (60.4)	1450 (55.0)	881 (54.5)	13787 (57.4)

a p < 0.001 comparing mapping status by laboratory setting
